# In vitro studies and in silico predictions of fluconazole and CYP2C9 genetic polymorphism impact on siponimod metabolism and pharmacokinetics

**DOI:** 10.1007/s00228-017-2404-2

**Published:** 2017-12-22

**Authors:** Yi Jin, Hubert Borell, Anne Gardin, Mike Ufer, Felix Huth, Gian Camenisch

**Affiliations:** PK Sciences, Novartis Institutes for Biomedical Research (NIBR), Fabrikstrasse 14, 202.3, CH-4002 Basel, Switzerland

**Keywords:** Siponimod, Pharmacokinetics, CYP2C9 polymorphism, SimCYP prediction, Fluconazole

## Abstract

**Purpose:**

The purpose of the study is to investigate the enzyme(s) responsible for siponimod metabolism and to predict the inhibitory effects of fluconazole as well as the impact of cytochrome P450 (CYP) 2C9 genetic polymorphism on siponimod pharmacokinetics (PK) and metabolism.

**Methods:**

In vitro metabolism studies were conducted using human liver microsomes (HLM), and enzyme phenotyping was assessed using a correlation analysis method. SimCYP, a physiologically based PK model, was developed and used to predict the effects of fluconazole and CYP2C9 genetic polymorphism on siponimod metabolism. Primary PK parameters were generated using the SimCYP and WinNonlin software.

**Results:**

Correlation analysis suggested that CYP2C9 is the main enzyme responsible for siponimod metabolism in humans. Compared with the CYP2C9*1/*1 genotype, HLM incubations from CYP2C9*3/*3 and CYP2C9*2/*2 donors showed ~ 10- and 3-fold decrease in siponimod metabolism, respectively. Simulations of enzyme contribution predicted that in the CYP2C9*1/*1 genotype, CYP2C9 is predominantly responsible for siponimod metabolism (~ 81%), whereas in the CYP2C9*3/*3 genotype, its contribution is reduced to 11%. The predicted exposure increase of siponimod with fluconazole 200 mg was 2.0–2.4-fold for CYP2C9*1/*1 genotype. In context of single dosing, the predicted mean area under the curve (AUC) is 2.7-, 3.0- and 4.5-fold higher in the CYP2C9*2/*2, CYP2C9*2/*3 and CYP2C9*3/*3 genotypes, respectively, compared with the CYP2C9*1/*1 genotype.

**Conclusion:**

.Enzyme phenotyping with correlation analysis confirmed the predominant role of CYP2C9 in the biotransformation of siponimod and demonstrated the functional consequence of CYP2C9 genetic polymorphism on siponimod metabolism. Simulation of fluconazole inhibition closely predicted a 2-fold AUC change (ratio within ~ 20% deviation) to the observed value. In silico simulation predicted a significant reduction in siponimod clearance in the CYP2C9*2/*2 and CYP2C9*3/*3 genotypes based on the in vitro metabolism data; the predicted exposure was close (within 30%) to the observed results for the CYP2C9*2/*3 and CYP2C9*3/*3 genotypes.

**Electronic supplementary material:**

The online version of this article (10.1007/s00228-017-2404-2) contains supplementary material, which is available to authorized users.

## Introduction

Siponimod (BAF312; Novartis Pharma AG, Basel, Switzerland) is an orally active, selective sphingosine 1-phosphate (S1P_1,5_) receptor modulator that is currently in clinical development for the treatment of relapsing multiple sclerosis with progressive disease [1]. S1P receptors play a key role in the physiological processes of a wide variety of cells, including those of the immune system and central nervous system (CNS) [2]. Preclinical studies have shown that siponimod binds to S1P_1_ and S1P_5_ receptors with nanomolar affinity [1]. Modulation of S1P_1_ receptors expressed on lymphocytes results in a prolonged internalisation of these receptors, which in turn prevents the egression of autoreactive lymphocytes from the lymph nodes and their recirculation to the CNS. In addition, modulation of S1P_5_ receptors expressed on oligodendrocytes promotes CNS repair mechanisms [2, 3].

The clinical pharmacokinetics (PK) of siponimod is linear in the dose range of 0.1 to 75 mg with clearance values ranged 3–4 L/h. Siponimod is mainly eliminated by oxidative metabolism in humans, based on kinetic results with recombinant human cytochrome P450 (CYP) and chemical inhibition experiments [unpublished data]. Owing to the predominant contribution of CYP2C9, it is likely that biotransformation of siponimod may be modified by CYP2C9 inhibitors and inducers, resulting in changes in siponimod exposure and pharmacological effects. To assess the effect of CYP2C9 inhibitors on siponimod metabolism, a drug–drug interaction (DDI) study with fluconazole was conducted as part of the clinical development programme. According to the regulatory guidelines, fluconazole, a moderate CYP2C9 inhibitor, is the recommended inhibitor for studies assessing CYP2C9 inhibition potential [4, 5].

Furthermore, the CYP2C9 enzyme is subject to significant genetic polymorphisms that vary largely among different ethnic populations [6]. The six well-known clinically relevant genotype variants of CYP2C9, out of 60 known alleles, are CYP2C9*1/*1, CYP2C9*1/*2, CYP2C9*1/*3, CYP2C9*2/*2, CYP2C9*2/*3 and CYP2C9*3/*3 [7]. CYP2C9*1 is the wild-type variant of the CYP2C9 polymorphic family and is the most common allele found in most populations, whereas CYP2C9*2 (11%) and CYP2C9*3 (7%) are more frequent in Caucasians only [8–10]. Three of the CYP2C9 polymorphic variants, CYP2C9*2/*2, CYP2C9*2/*3 and CYP2C9*3/*3, may lead to clinically relevant reductions in enzyme activity, and patients with these alleles have been designated as ‘poor metabolisers (PMs)’ [7, 11]. Thus, increased plasma exposure of siponimod is possible in subjects with CYP2C9 PM genotypes.

Accurate predictions of human PK will help in designing of clinical trials and understanding the absorption, distribution, metabolism and excretion (ADME) properties of compounds. This in turn may enable early selection of the best candidate drugs for development. Use of physiologically based PK (PBPK) modelling and simulation methods has been receiving increased attention in drug discovery and development [12, 13] and in submissions for regulatory filing [14, 15].

Simulation methods such as the SimCYP (SimCYP Ltd., Sheffield, UK) population-based ADME simulator help better understand the mechanisms that determine siponimod disposition in vivo. The simulator combines data generated during the preclinical stage and data from in vitro studies with relevant physicochemical attributes of a compound and dosage forms with demographic, physiological and genetic information on different patient populations to predict in vivo PK parameters and profiles [16]. SimCYP simulations were conducted to predict the exposure change of siponimod under CYP2C9 inhibition (fluconazole) in healthy subjects with the CYP2C9*1/*1 genotype and to evaluate the impact of CYP2C9 genetic polymorphism on systemic exposure of siponimod in subjects with varying homozygous and heterozygous CYP2C9 genotypes.

Here, we report (i) the contribution of CYP2C9 in the metabolism of siponimod by correlation analysis and functional consequence of genetic polymorphism on siponimod metabolism by using individual human liver microsomes (HLM) in vitro, (ii) the prediction of inhibitory effects of fluconazole on siponimod exposure with SimCYP simulation and (iii) the prediction of the effects of various CYP2C9 genotypes on siponimod PK by using SimCYP simulation.

## Materials and methods

### In vitro study using HLM

#### Biological materials

Single-donor HLM from three homozygous CYP2C9 genotypes (donor HG6, CYP2C9*1/*1; donor HG103, CYP2C9*2/*2; and donor HK27, CYP2C9*3/*3) were purchased from BD Biosciences (Woburn, MA, USA). For correlation analysis of CYP reaction phenotyping, a kit of individual liver microsomes from 16 donors was purchased from XenoTech, LLC (Lenexa, KS, USA), and the correlation analysis was performed as described [17]. Details on chemicals and reagents are described in Online Resource 1.

#### Radiolabelled drug

**Fig. 1:**
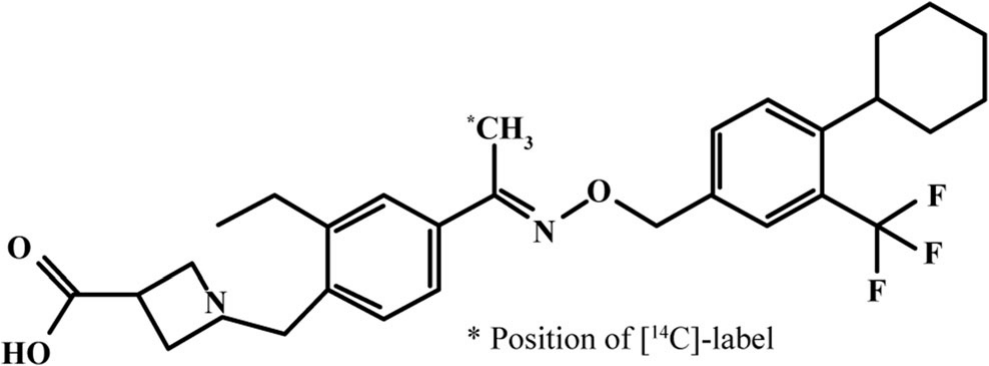
Chemical structure of [^14^C]siponimod

The radiolabelled [^14^C]siponimod hemifumarate was synthesised by the Isotope Laboratory of Novartis (Basel, Switzerland). Radiochemical purity of the drug was 98.9%, with a specific activity of 4.218 MBq/mg [^14^C]siponimod free base. The chemical structure of the radiolabelled siponimod is shown in Fig. 1.

#### Incubation of HLM

HLM were incubated in 0.1 M Tris buffer, pH 7.4, at 37 °C. Typically, the final incubation volume was 200 μL, and the total volumes were prepared as follows: 10 μL of 100 mM MgCl_2_, stock substrate solutions with 0.25 CHAPS (to increase solubility), and microsomes were added to the appropriate volume of the buffer. Reaction was started by adding 20 μL of a fresh 10 mM nicotinamide adenine dinucleotide phosphate (NADPH) (1 mM, final concentration). All the in vitro experiments were carried out as duplicates. The final concentration of siponimod used in vitro was 5 μM. After 60 min of incubation, the reactions were terminated by adding ice-cold 0.5% formic acid in acetonitrile, kept for 30 min at − 80 °C (or overnight at − 20 °C) and followed by centrifugation at 30,000×*g* for 15 min to remove proteins. For the experiments, aliquots of the supernatants were analysed using high-performance liquid chromatography (HPLC) combined with radioactivity detector. The metabolism of [^14^C]siponimod was studied in liver microsomes from individual donors with the CYP2C9*1/*1, CYP2C9*2/*2 and CYP2C9*3/*3 genotypes to investigate the functional consequence of CYP2C9 genetic polymorphism on siponimod metabolism. Formation of metabolites was quantified by HPLC combined with radioactivity detection.

#### Correlation analysis and genotype sensitivity analysis on the functional consequences on in vitro metabolism

The correlation analysis of siponimod metabolism was performed using HLM from 16 individual donors obtained from XenoTech. The microsomes were characterised by the manufacturer for their catalytic activities of CYP-specific enzymes. The incubation time was 60 min, and HLM concentration was 0.1 mg/mL. The potential correlation between total [^14^C]siponimod metabolism in HLM from single donors and the reported activities for different CYP isoforms were analysed by linear regression analysis using Microsoft Excel (version 2002, Microsoft Corporation, Redmond, WA, USA). In vitro metabolic activity of different CYP2C9 isoform genotypes was determined from in vitro metabolism data for homozygote and by linear extrapolation between the homozygous for the heterozygote CYP2C9 genotypes.

Details on HPLC with radiodetection method used in the study are provided in the Online Resource **1**.

### SimCYP model to predict siponimod exposure with concomitant CYP2C9 inhibitor (fluconazole)

After trying different modelling approaches, a SimCYP simulation model was optimised to predict exposure changes of siponimod when co-administered with fluconazole in healthy subjects with the CYP2C9*1/*1 genotype. The input parameters used for developing the model are summarised in the Online Resource 2. The simulations were performed using the SimCYP population-based ADME Simulator (versions 9 and 16, SimCYP Ltd., Sheffield, UK). All input parameters for the fluconazole model were used according to SimCYP V9, except for the intravenous CL (CL_iv_) value, which was taken from the previous SimCYP version 7 (Online Resource 3). In SimCYP simulation V16, the CYP2C9 *K*_*i*_ value of 20.4 μM for fluconazole was adapted based on published data [18] and a model verified by Certara [unpublished data]. In vitro and in vivo inputs were combined to optimise PK simulations. For PK parameters, absorption was predicted using the first-order model, and distribution was simulated using the minimal PBPK model. The SimCYP retrograde model was applied for the simulation of siponimod PK by using the mean oral clearance (CL/F) from different single-dose administrations. Time-based simulations were performed to predict PK parameters and profiles of siponimod with and without the co-administration of fluconazole.

SimCYP prediction was performed in 70 healthy subjects (14 × 5 arms) for the first three dosage regimens with the CYP2C9*1/*1 genotype. The dosage regimens administered in the following trials were (i) fluconazole 200 mg once a day (qd) from day 1 to day 20 and siponimod 5 mg on day 4, (ii) fluconazole 200 mg twice a day (bid) on day 1 and 200 mg qd from day 2 to day 21 and siponimod 5 mg on day 3, (iii) fluconazole 400 mg bid on day 1 and 400 mg qd from day 2 to day 19 and siponimod 4 mg on day 3, (iv) fluconazole 200 mg bid on day 1 and 200 mg qd from day 2 to day 19 and siponimod 4 mg on day 3 by using a refined model in SimCYP V16, reflecting the design of the clinical DDI study.

#### Data analysis

PK parameters were calculated, and statistical analyses were performed using a SimCYP simulator (V9 and 16). All the simulated areas under the concentration–time curve (AUC) values were calculated up to the last time point (AUC_last_) by using the SimCYP simulation software. The half-life (*T*_1/2_) was determined using non-compartmental methods (WinNonlin Pro version 5, Pharsight Corporation, CA, USA).

### SimCYP simulation model to predict siponimod PK in a population with CYP2C9 genetic polymorphism

The SimCYP simulator incorporated a physiologically based method to simulate changes in CL associated with various CYP2C9 genotypes. In vitro and PK data on siponimod were collected from internal siponimod studies to run the simulation model (SimCYP V9, V12 and V16; Online Resource 2). The enzyme contribution for siponimod metabolism in the different CYP2C9 genotypes was predicted from in vitro enzyme phenotyping [unpublished data], and metabolism data derived from HLM homozygotes were used to predict the CPY2C9 intrinsic clearance (CL_int_). For the heterozygote genotypes, CL_int_ of CYP2C9 was estimated through linear extrapolation of the values from homozygote genotypes (Table 1).

**Table 1:** Intrinsic clearance of allelic CYP2C9 genotypes relative to wild-type genotype (CYP2C9*1/*1) for SimCYP model construction

CYP2C9 genotype	In vitro PG ratios^a^	CL_int_ (μL/min/pmol)^b^
CYP2C9*1/*1	1.0 ± 0.062	49.07 ± 3.04
CYP2C9*1/*2	0.673	33.03
CYP2C9*1/*3	0.5445	26.72
CYP2C9*2/*2	0.345 ± 0.006	16.93 ± 0.29
CYP2C9*2/*3	0.217	10.65
CYP2C9*3/*3	0.089 ± 0.023	4.37 ± 1.14

Student’s *t* tests are applied for comparing the significant difference of CYP2C9*1/*1 to CYP2C9*2/*2 (*p* value 0.0477) and CYP2C9*1/*1 to CYP2C9*3/*3 (*p* value 0.0424), respectively

*CYP* cytochrome P450, *PG* pharmacogenetics

^a^The in vitro PG ratio data is from human liver microsomes

^b^The intrinsic clearance in the different genotypes was estimated by multiplying the in vitro PG ratio with the intrinsic clearance in the wild-type genotype (49.07 μL/min/pmol) simulated from the SimCYP (V12) retrograde model

The simulations were performed in the SimCYP virtual healthy subject population aged 18–65 years (50% women, and 3 groups of 15 virtual healthy subjects each, *n* = 45) with CYP2C9 genetic variants CYP2C9*1/*1, CYP2C9*1/*2, CYP2C9*2/*2 and CYP2C9*3/*3. Simulation with the CYP2C9*1/*3 and CYP2C9*2/*3 genotypes was performed together with 25 individuals in each population group.

A retrograde model for the wild-type (WT) genotype was applied for clearance simulation of siponimod by using the mean of median clearance from different single doses. Enzyme contribution ratios were obtained from in vitro CYP phenotyping experiments [unpublished data]. Intrinsic clearance of allelic CYP2C9 variants was calculated relative to WT values based on the metabolism ratio determined in vitro (PG ratios), as presented in Table 1. The mean enzymatic activities and CL_int_ of other enzymes were assumed unchanged (independent) with the CYP2C9 genotypes. Further information about the SimCYP PBPK model method development is detailed in Online Resource 4.

## Results

### In vitro study using HLM

#### Correlation analysis in HLM

In vitro metabolism of [^14^C]siponimod was investigated in HLM from 16 individual donors to analyse their ability to metabolise siponimod. The correlation analysis of [^14^C]siponimod total metabolism with various enzyme marker activities is presented in Table 2. The best correlation was shown for CYP2C9, with correlation coefficients (*R*) of 0.705 to 0.719 with diclofenac 4′-hydroxylation activity, whereas the other CYP-specific marker activities scored lower correlation values and thus indicate no significant correlation with [^14^C]siponimod metabolism in these microsomes.

**Table 2:** Correlation of siponimod metabolism with the sample-to-sample variation in various marker enzyme activities in a bank of individual human liver microsomes

Linear regression correlation coefficient (*R*)						
P450-specific marker reaction	Enzyme	Siponimod depletion	M5	M6	M7	Formation of all metabolites
7-Ethoxyresorufin O-dealkylation	CYP1A2	− 0.281	− 0.251	− 0.181	− 0.217	− 0.233
Coumarin 7-hydroxylation	CYP2A6	0.251	0.226	0.062	0.248	0.222
S-mephenytoin *N*-demethylation	CYP2B6	− 0.036	0.548	0.712	0.604	0.603
Paclitaxel 6α-hydroxylation	CYP2C8	0.527	0.513	0.668	0.542	0.554
Diclofenac 4′-hydroxylation	CYP2C9	0.719	0.716	0.530	0.706	0.705
S-mephenytoin 4′-hydroxylation	CYP2C19	0.005	− 0.006	0.160	− 0.032	0.001
Dextromethorphan O-demethylation	CYP2D6	0.246	0.235	0.096	0.263	0.236
Chlorzoxazone 6-hydroxylation	CYP2E1	0.377	0.387	0.354	0.415	0.403
Testosterone 6β-hydroxylation	CYP3A4/5	0.398	0.358	0.523	0.358	0.384
Lauric acid 12-hyroxylation	CYP4A11	− 0.036	0.034	0.013	0.018	0.025

M5, M6 and M7 are metabolites of siponimod [unpublished data]

*CYP* cytochrome P450

**Fig. 2:**
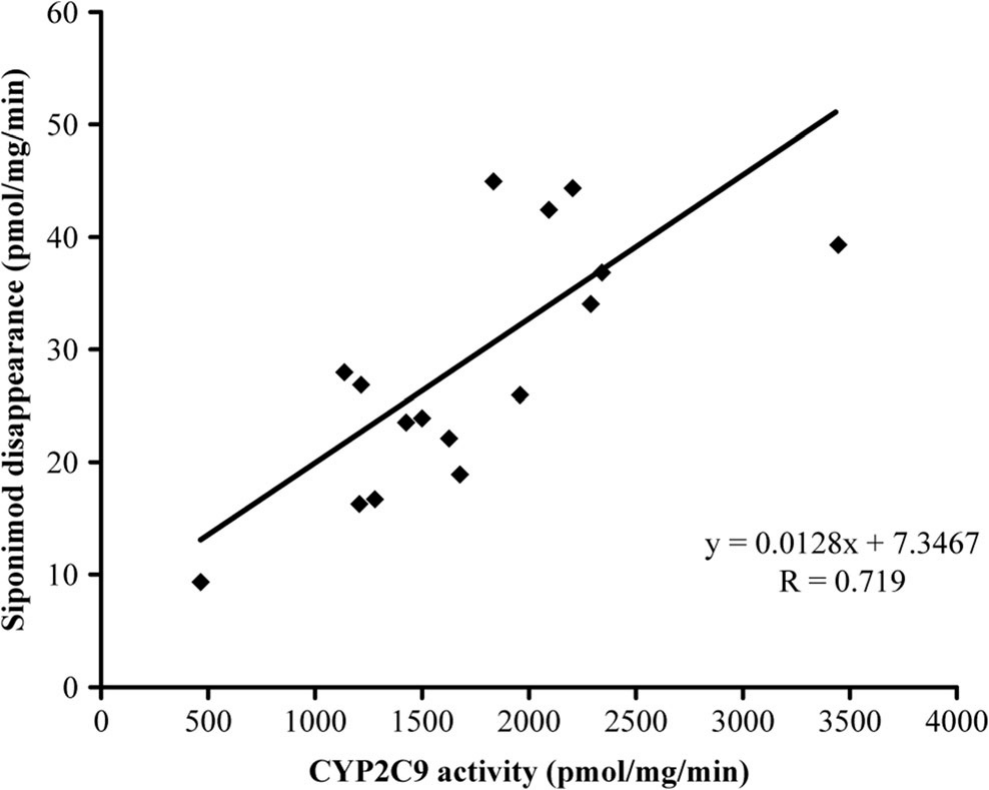
In vitro correlation analysis of [^14^C]siponimod metabolism rates with CYP2C9 enzyme activities. CYP cytochrome P450

In vitro correlation of diclofenac 4′-hydroxylation activity was obtained with the formation of the major metabolites M5 and M7 with *R* > 0.7 (Table 2). The correlation results confirmed that metabolites M5 and M7 are mainly catalysed by CYP2C9 in HLM. The results of the correlation analysis indicated that CYP2C9 was the major enzyme involved in the hepatic oxidative metabolism of [^14^C]siponimod (Fig. 2, Table 2).

#### In vitro sensitivity analysis and functional consequence in different CYP2C9 genotypes

**Fig. 3:**
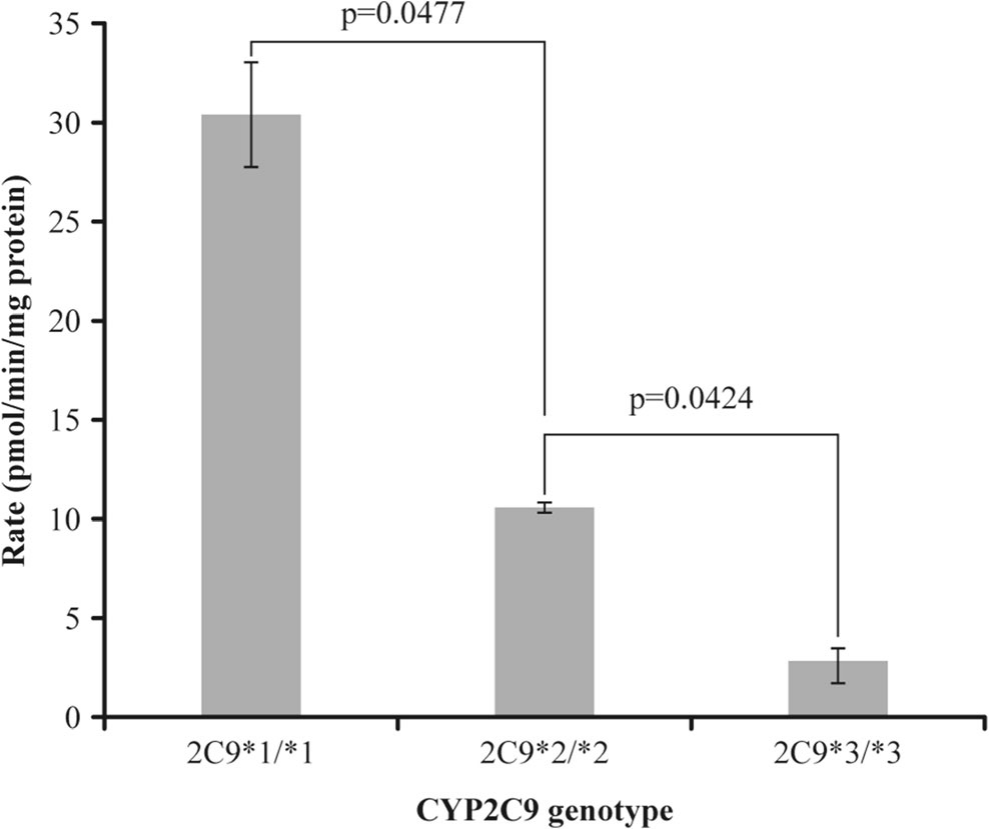
Comparison of [^14^C]siponimod metabolism rates in human liver microsomes (HLM) from individual donors with three different CYP2C9 genotypes. Formation of total metabolites was quantified by HPLC with radioactivity detection. Student’s *t* tests are applied for comparing the significant difference of CYP2C9*1/*1 to CYP2C9*2/*2 (*p* value 0.0477) and CYP2C9*1/*1 to CYP2C9*3/*3 (*p* value 0.0424), respectively. CYP cytochrome P450

Compared with CYP2C9*1/*1, the metabolic rates of siponimod in HLM from CYP2C9*2/*2 and CYP2C9*3/*3 donors were substantially lower (Fig. 3). In the CYP2C9-genotyped individual HLM, an approximately 3-fold decrease in the rate of hydroxylated metabolite formation was evident in the CYP2C9*2/*2 samples compared with the CYP2C9*1/*1 samples. A greater reduction (up to 10-fold) in the formation of hydroxylated metabolite in the liver microsomal sample with CYP2C9*3/*3 genotype was observed. These results confirm the potential role of genetic polymorphism with functional consequence in the oxidative metabolism of siponimod.

**Fig. 4:**
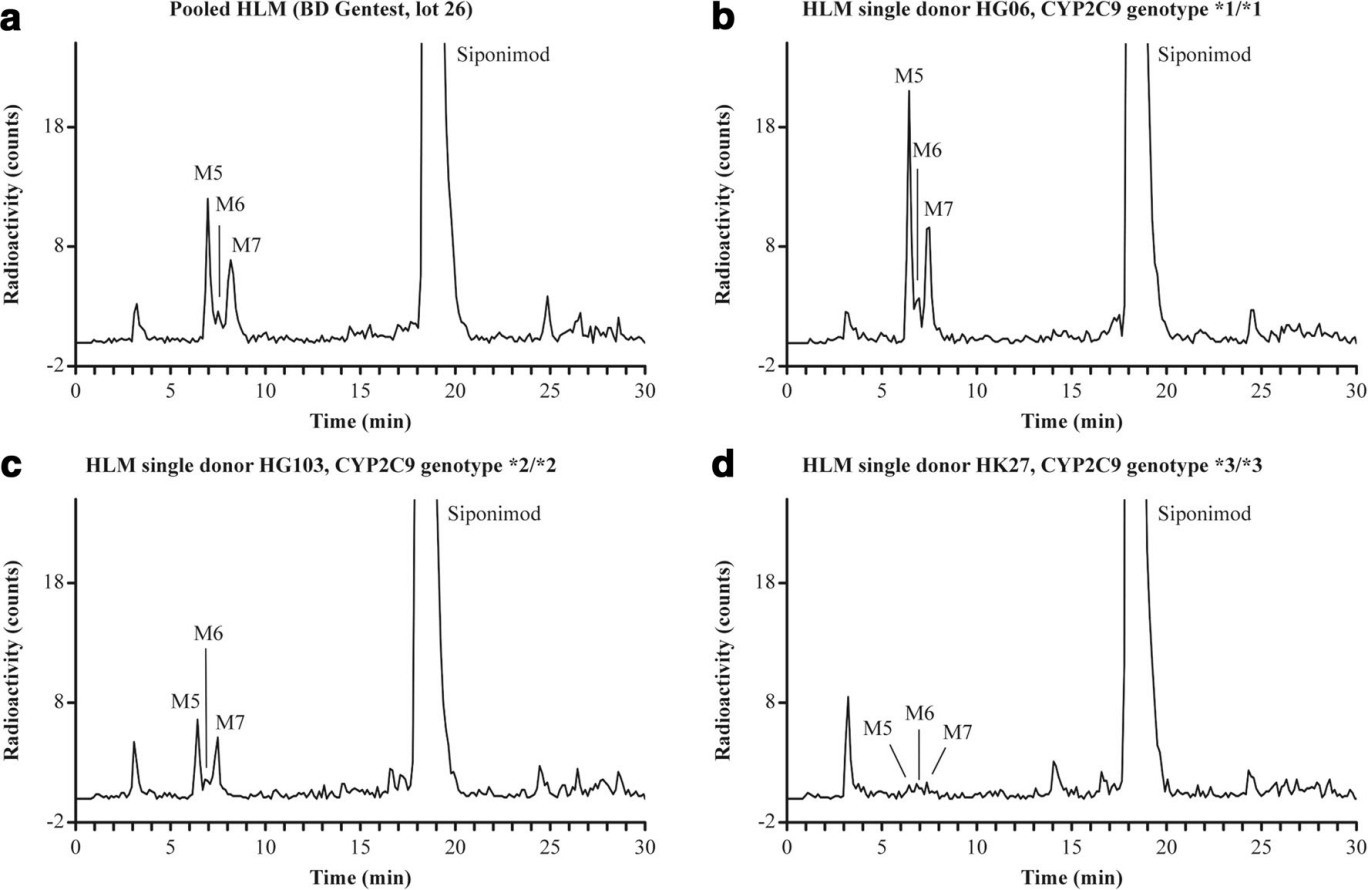
Chromatogram of [^14^C]siponimod after incubation with human liver microsomes of three CYP2C9 genotypes and comparison with pooled human liver microsomes. Five micromolar siponimod was incubated with 1 mg/mL human liver microsomes for 60 min at 37 °C. Siponimod and metabolites were monitored by HPLC with radioactivity detection. CYP cytochrome P450, HPLC high-performance liquid chromatography, HLM human liver microsomes

The metabolic pattern of siponimod in a CYP2C9*1/*1 donor was similar to that in the pool of HLM with M5 and M7 as major metabolites. In the CYP2C9*2/*2 donors, M5 and M7 decreased significantly, whereas in the CYP2C9*3/*3 donors, very strong reduction of metabolites was observed (Fig. 4).

Owing to this functional consequence of CYP2C9 genetic polymorphism demonstrated with siponimod as a substrate, variable metabolic capacity can be expected in individuals with different genotypes.

### Prediction of siponimod exposure changes by fluconazole inhibition

Siponimod absorption was predicted based on the permeability of Caco-2 cell (human colon carcinoma cell line) monolayers and considered to be complete (fraction absorbed [fa] = 1). Absorption rate constant (Ka) was calculated from the first clinical data to be 0.98/h. Owing to the low plasma unbound fraction (fu), fugut was assumed to be low as well and set to 0.0002 (=fu), resulting in a fraction escaping gut clearance (fg) of 1.0. A volume of distribution of 2.12 L/kg was estimated based on preclinical data observation. The initial CL estimation of the siponimod model was based on human in vitro data from HLM and recombinant enzyme kinetics [unpublished data]. By using the in vivo clearance (CL/F) together with the fractional contribution of each CYP enzyme (phenotyping data) in the SimCYP retrograde calculator, the enzyme CL_int_ values were estimated, resulting in an improved drug exposure prediction (Table 3).

**Table 3:** Predictions of fluconazole inhibition on siponimod pharmacokinetics using SimCYP ADME simulator version 9 (i, ii, iii) or version 16 (iv) and comparison with observed in vivo results

Trial no. (dose regimen)	Fluconazole		Siponimod	AUC/D (h ng/mL)/mg	AUC (h ng/mL)	CL/F (L/h)	AUC_*i*_/AUC	*C*_max_,_*i*_/*C*_max_
	Day 1	Day 2 till the last day						
i	200 mg qd	200 mg qd	5 mg, day 4	249	1245	4.01	1.97	1.09
ii	200 mg bid	200 mg qd	5 mg, day 3	255	1273	3.93	2.43	1.10
iii	400 mg bid	400 mg qd	4 mg, day 3	257	1028	3.89	3.42	1.11
iv	200 mg bid	200 mg qd	4 mg, day 3	272	1089	3.71	2.15	1.07
in vivo*	200 mg bid	200 mg qd	4 mg, day 3	280	1110	3.59	1.97	1.10

Siponimod-predicted PK parameters are geometric mean of 70 virtual individuals; SimCYP version 9 was used for trials i to iii, and version 16 was used for trial iv. The CV% of the in vivo study is reported in detail in Table 2 of the companion manuscript (EJCP-17-D-17-00482). The CV ranges for the four simulations are *C*_max_ ratio (2–5%), AUC ratio (10–23%), CL/F (45–60%), AUC (41–59%) and AUC/D (41–59%), respectively

*AUC* area under the concentration–time curve, *AUC*_*i*_ area under the concentration–time curve under fluconazole inhibition, *bid* twice a day, *CL/F* apparent systemic clearance, *C*_*max*_ maximum plasma concentration, *D* dose, *qd* once a day, *T*_*max*_ time to maximum plasma concentration, *T*_*1/2*_ terminal half-life

**Fig. 5:**
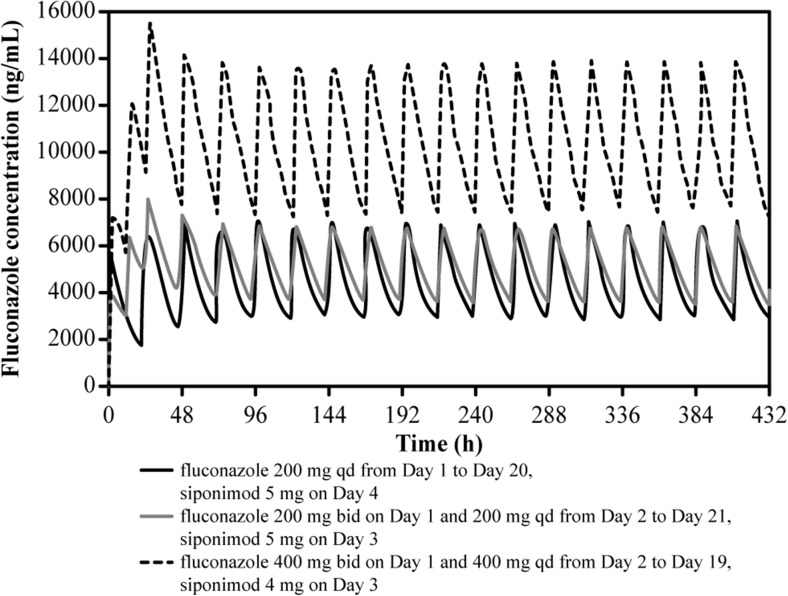
Simulated mean plasma concentration of fluconazole over time using SimCYP (version 9). bid twice a day, h hours, qd once a day

Based on the simulated profile, the fluconazole bid regimen (on day 1) achieved maximum plasma concentration (*C*_max_) levels more rapidly than did the fluconazole qd regimen (Fig. 5). Steady-state fluconazole concentrations (dose 200 mg) were reached more rapidly for optimal inhibition in less time when the trial ii dose regimen was administered (Table 3).

**Fig. 6:**
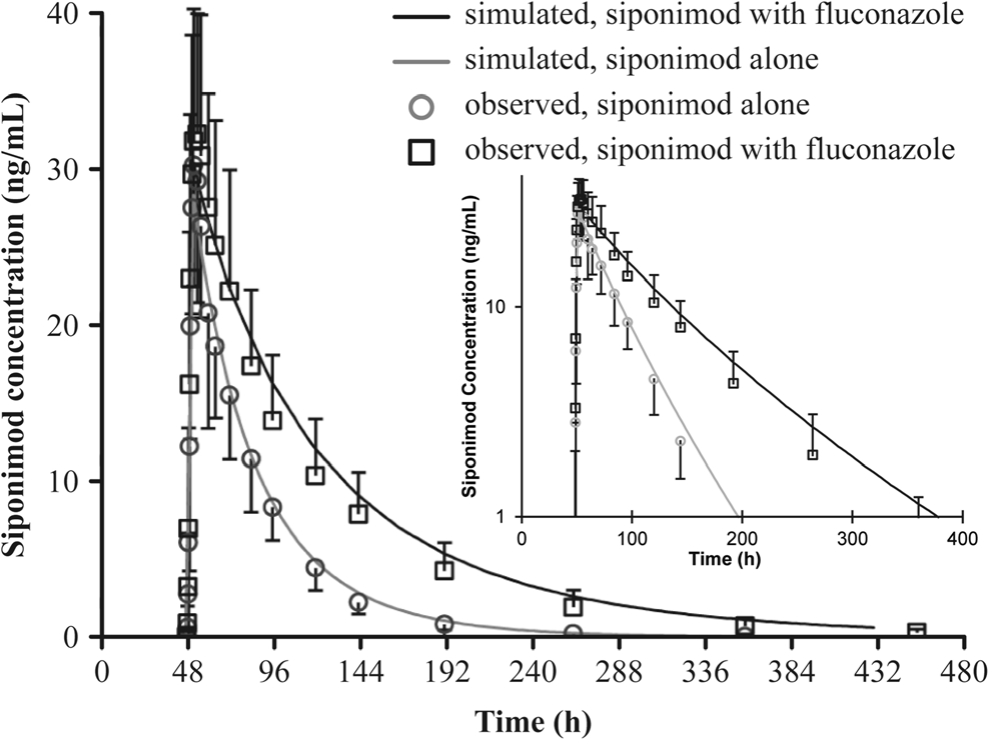
Predicted siponimod mean plasma concentration in the absence and presence of fluconazole (200 mg) inhibition (dose regimen iv, SimCYP version 16). Observed data (*n* = 14 for siponimod alone; *n* = 11 for siponimod with fluconazole) are included for comparison. The SimCYP simulation was performed in 110 virtual subjects. h hours

The predicted geometric mean AUC_last_/AUC ratio of siponimod with different dose regimens of fluconazole ranged from 1.97 (trial i) to 2.43 (trial ii) with fluconazole 200 mg. When the daily dose of fluconazole was increased to 400 mg from day 2 (trial iii), the predicted geometric mean AUC ratio of siponimod increased to 3.42 (Table 3). In general, across all trials, the predicted *C*_max_ ratio was approximately 1.1, and the predicted mean CL of siponimod was approximately 4 L/h (Table 3). Using the last SimCYP version 16 (trial iv), an AUC ratio of 2.15 and an increase in siponimod *T*_1/2_ from 26 to 49 h was predicted (Fig. 6, Table 3) for the clinical fluconazole inhibition study. The PK profile and parameters were very close to the observed clinical results.

### Prediction of siponimod metabolism and PK in genetic polymorphic CYP2C9 populations

Prediction of hepatic enzyme contribution in populations with polymorphic CYP2C9 genotypes is presented in Table 4. In the CYP2C9*1/*1 genotype population, CPY2C9 showed a predominant contribution of 81%. The CYP2C9 contribution decreased progressively to 11% in the CYP2C9*3/*3 population (PM), whereas the relative contribution by CYP3A4 increased from 17 to 79%.

**Table 4:** Predicted contribution ratio of CYP2C9 and other P-450 enzymes for siponimod human metabolism

Genotype	Predicted enzyme contribution ratio (% fm)				
	CYP2B6	CYP2C8	CYP2C9	CYP2C19	CYP3A4
CYP2C9*1/*1	0.24	1.54	80.84	0.15	17.23
CYP2C9*1/*2	0.47	2.54	68.83	0.23	27.94
CYP2C9*1/*3	0.49	3.36	62.53	0.30	33.32
CYP2C9*2/*2	0.60	3.48	57.81	0.33	37.78
CYP2C9*2/*3	0.83	4.81	43.84	0.43	50.09
CYP2C9*3/*3	1.28	7.74	11.31	0.76	78.91

*CYP* cytochrome P450, *fm* fraction metabolised

**Fig. 7:**
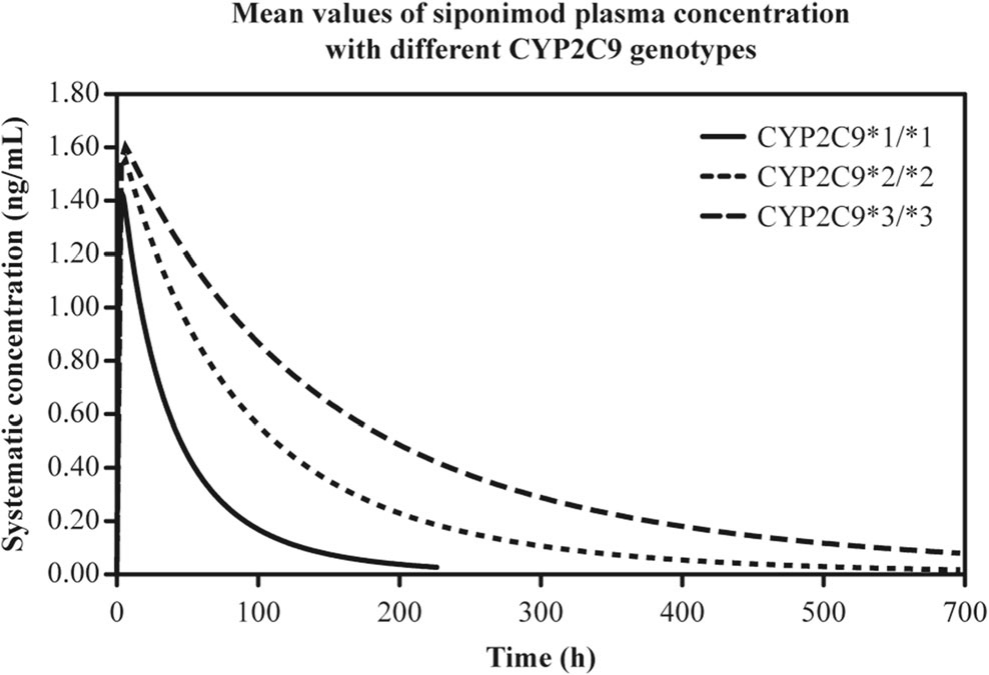
Simulated mean plasma concentration of siponimod after single oral dose of 0.25 mg in the genetic polymorphic population of homozygote CYP2C9 genotypes using SimCYP version 12. Only simulated profiles of the three homozygotes genotypes are shown. CYP cytochrome P450, h hours

Simulated plasma concentration–time profiles and predicted PK parameters after administration of a single oral dose of siponimod 0.25 mg across different genetic polymorphic CYP2C9 populations are presented in Fig. 7 and Table 5. Compared with the CYP2C9*1/*1 genotype, a 2.7-fold, 3.0-fold and 4.5-fold increase in the geometric mean AUC of siponimod was predicted in the CYP2C9*2/*2, CYP2C9*2/*3 and CYP2C9*3/*3 genotypes, respectively.

**Table 5:** Prediction of pharmacokinetic parameters in different CYP2C9 genotype sub-populations using SimCYP (version 12) simulator, following administration of siponimod (0.25 mg single oral dose)

CYP2C9 genotype	*1/*1	*1/*2	*2/*2	*2/*3^a^	*3/*3
AUC_last_ (ng/mL h)	62.3	90.0	167.5	185.0^b^	278.7
AUC ratio to wt	1.0	1.4^b^	2.7	3.0^a^	4.5
Median *T*_max_ (h)	4.22	4.92	5.37	6.17	5.92
*T*_1/2_ (h)	33	42	78	104	170
*C*_max_ (μg/mL)	1.32	1.37	1.44	1.37	1.47
Dose (mg)	0.25	0.25	0.25	0.25	0.25
CL/F (L/h)	4.01	2.78	1.49	1.35	0.90

All values are geometric mean unless specified otherwise

*AUC*_*last*_ area under the plasma concentration–time curve from time zero to the time of the last quantifiable concentration, *CL/F* apparent systemic clearance, *C*_*max*_ maximum plasma concentration, *CYP* cytochrome P450, *T*_*max*_ time to maximum plasma concentration, *T*_*1/2*_ terminal half-life, *wt* wild type

^a, b^Median value

The elimination *T*_1/2_ increased up to 5-fold from 33 h in subjects with CYP2C9*1/*1 genotype to 104 h in CYP2C9*2/*3 genotype and 170 h in CYP2C9*3/*3 genotype. Subjects with heterozygous alleles showed an increase in geometric mean AUC of 1.4 for CYP2C9*1/*2 and a median AUC increase of 3.0- and 2.1-fold for the CYP2C9*2/*3 and CYP2C9*1/*3 genotypes, respectively.

## Discussion

Siponimod is mainly eliminated by oxidative metabolism with CYP2C9 as the predominant hepatic enzyme [unpublished data]. HLM provide one of the most convenient ways to explore in vitro metabolic properties and provide a useful approach to predict human drug metabolic profiles in vivo. Simulation using PBPK modelling is a useful tool to predict drug mechanisms, behaviour and ADME profiles before investigation in an in vivo study [16]. This approach reduces the failure rate in the drug development process. SimCYP incorporates a physiologically based simulation model to predict changes in inhibitor and substrate concentrations over time and generation of inhibitory metabolites. Further, it also aids to predict inhibition of hepatic and gastrointestinal metabolism, active uptake of the substance into the liver and the effect of population variability [16].

Results of the correlation analysis indicated CYP2C9 as the major enzyme involved in the hepatic oxidative metabolism of [^14^C]siponimod. CYP2C9 mainly produced the metabolites M5 and M7. Both can therefore be considered as CYP2C9 selective or specific metabolites of siponimod [unpublished data]. A significant correlation was observed between siponimod metabolites (M5 and M7) and diclofenac 4′-hydroxylation activity, confirming that the formation of metabolites M5 and M7 is mainly catalysed by CYP2C9 in HLM. Enzyme phenotyping was traditionally performed using three basic approaches (chemical inhibition, recombinant enzymes and correlation analysis) documented in the scientific literature [19] and recognised by health authorities. Each of these approaches has its advantages and shortcomings, and so, a combination of methods is essential. For siponimod, the results of this enzyme phenotyping approach (correlation analysis) are in line with and confirm the results obtained using chemical inhibition and recombinant enzymes [unpublished data] and therefore provide further support and additional confidence to the key contribution of CYP2C9 to siponimod metabolism. The metabolic pattern of siponimod in CYP2C9*1/*1 HLM donor was similar to that in the pool of HLM with M5 and M7 as the major metabolites. In the CYP2C9*2/*2 HLM donors, M5 and M7 formation was significantly reduced, whereas practically no metabolites were detected in HLM with the CYP2C9*3/*3 genotype.

We have started with a bottom-up approach by using in vitro human data to construct the PBPK model. For PK predictions, absorption was predicted using the first-order model after trying prediction with in vitro Caco-2 permeability. Distribution was simulated using the minimal PBPK model initially and then switched to the full PBPK model in the V16 simulation. The retrograde model in the SimCYP software was finally selected for simulation of siponimod PK by using in vivo clearance (CL/F) combined with in vitro enzyme phenotyping experiments generating the fraction metabolised (fm) of major drug metabolising enzymes [unpublished data].

The improved SimCYP model was used to predict the AUC changes in siponimod when co-administered with different doses of the CYP2C9 inhibitor fluconazole. Fluconazole was selected because it is one of the most potent CYP2C9 inhibitors used in clinical medicine and is recommended in the regulatory guidance as a prototype inhibitor for use in human DDI studies for compounds that are predominantly metabolised by CYP2C9 [20].

In the SimCYP simulations, a mean AUC increase of 2- to 2.4-folds was predicted when siponimod was co-administered with fluconazole 200 mg. The trial ii fluconazole dose regimen was comparable to the findings of the clinical DDI study, which resulted in an in vivo AUC ratio of 1.97 and a *C*_max_ ratio of 1.1. The observed AUC ratio was about 20% lower than the previous prediction; however, the predicted and observed *C*_max_ ratios were almost identical or very similar. The predicted DDI effects using SimCYP simulations were confirmed in vivo*,* with a 20% deviation from the observed AUC ratio.

The SimCYP simulations predicted that siponimod *T*_1/2_ would be increased owing to inhibition of CYP450s (CYP2C9 and CYP3A4) by fluconazole. Accordingly, in the human study, the geometric mean *T*_1/2_ increased from 40.6 to 61.7 h (approximately 50%).

In the wild-type genotype CYP2C9*1/*1, CYP3A4 plays a minor role in siponimod metabolism with a contribution of 17% and no significant inhibition or induction effect is expected to this metabolic pathway. However in individuals with variant CYP2C9 alleles, due to the reduced enzymatic activities, the fractions metabolised by CYP2C9 decrease while the relative CYP3A4 contribution is significantly increased (Table 4), particularly in the case of CYP2C9*3/*3 genotype. We have also performed SimCYP predictions on effects of CYP3A inhibitors and inducer in individuals with variant CYP2C9 alleles [unpublished data]. Strong CYP3A4 inhibitors and inducers have potential to affect siponimod pharmacokinetics with variant CYP2C9 alleles, particularly in individuals with the CYP2C9*3/*3 genotype, which has the highest fractional CYP3A4 contribution.

A greater siponimod exposure was observed in CYP2C9*3 carriers than in those without the CYP2C9*3 genotype. Reduced CYP2C9 enzymatic activity in PMs was reported to prolong the elimination of siponimod, with no effect on the absorption phase of siponimod.

The relative difference between homozygotes in in vitro metabolism was used to construct a pharmacogenetic polymorphic model in SimCYP for siponimod. As no metabolism activity in the heterozygous donor HLM was available for measurement during in vitro studies, the pharmacogenetic ratios in the heterozygote genotypes were linearly extrapolated from the homozygote CYP2C9 genotypes. This approach might have some limitation in the quantitative prediction for the heterozygotic CYP2C9 population. Compared with the CYP2C9*1/*1 genotype, siponimod AUC was predicted to be 4.5-fold higher in subjects with the CYP2C9*3/*3 genotypes, and these findings were consistent with historical study data following single oral dose of siponimod 0.25 mg [unpublished data]. In the clinical study, CYP2C9*2/*3 and CYP2C9*3/*3 individuals experienced an approximately 2- and 4-fold higher drug exposure, respectively, than that experienced by the CYP2C9*1/*1 healthy subjects, which confirmed the predicted AUC increase. The AUC ratio of CYP2C9*3/*3 to the wild type was 4.5 for the prediction and 4.0 for the observed data, which indicates a difference of < 15% and falls within the bioequivalence range [21].

Human clearance data revealed good in vitro to in vivo correlation when using the in silico modelling predictions. Overall, the proposed application of the in vitro and in silico studies of CYP2C9 for siponimod represents a novel approach for estimation of PK parameters and population variability regarding CYP2C9 genetic polymorphism. Implementation of in vitro*–*in silico extrapolation techniques using SimCYP simulation yields a better understanding of potential DDIs and genetic polymorphism in humans.

## Conclusion

In conclusion, correlation analysis supported the results of the other two reaction phenotyping approaches and confirmed CYP2C9 as the major enzyme responsible for siponimod metabolism. With the use of individual genotyped HLM, the functional consequence of CYP2C9 genetic polymorphism was demonstrated in siponimod metabolism. Moreover, co-administration of a moderate CYP2C9 inhibitor (fluconazole) was predicted to result in a 2- to 2.4-fold increase in siponimod AUC. The good in vitro to in vivo extrapolation using SimCYP modelling shows the importance of using in vitro human metabolism data for in silico simulation to predict the fate of siponimod metabolism in early clinical studies with CYP2C9 inhibitors. The simulations of fluconazole inhibition and pharmacogenetic effects were performed during the planning phase of clinical protocols. The results of the prediction were used to optimally design the clinical study protocols. This example demonstrates that SimCYP simulation can assist the clinical protocol design and make a robust prediction of the clinical outcome for siponimod. SimCYP modelling has accurately predicted the in vivo siponimod PK in the CYP2C9 genetic polymorphic sub-populations. There is a considerable interethnic variation in the incidence of PM genotypes. The CYP2C9 genotype has a significant impact on siponimod metabolism both in vitro and in vivo. In individuals with certain genetic polymorphisms, the activity of this enzyme is reduced resulting in higher systemic exposure. Nevertheless, the clinical relevance of these observations remains to be established. The functional consequence of CYP2C9 polymorphism with siponimod as a substrate observed both in vitro and in vivo and predicted through SimCYP PBPK simulation suggests the possibility of therapeutic dose adjustment based on patient genotyping for personalised medicine, with the potential to provide better treatment to closely match the individual condition of patients.

## Electronic supplementary material

Online Resource 1 (DOCX 14 kb)

Online Resource 2 (DOCX 20 kb)

Online Resource 3 (DOCX 20 kb)

Online Resource 4 (DOCX 24 kb)
