# Supplementary material for: In vitro studies and in silico predictions of fluconazole and CYP2C9 genetic polymorphism impact on siponimod metabolism and pharmacokinetics
Source: Eur J Clin Pharmacol. 2017 Dec 22;74(4):455–64. doi: 10.1007/s00228-017-2404-2 (PMC5849655; doi:10.1007/s00228-017-2404-2)
Supplement: Supplementary file 1 — (DOCX 14 kb) [file 228_2017_2404_MOESM1_ESM.docx]

**Supplementary materials**

**Online Resource 1 .** Aditional materials and methods

**Chemicals and reagents:** Trifluoroacetic acid and water were obtained from Fluka AG (Buchs, Switzerland). Formic acid and acetonitrile were obtained from Merck (Darmstadt, Germany). β-nicotinamide adenine dinucleotide phosphate (β-NADPH) was procured from Sigma (St Louis, MO, USA), and Tris buffer (pH 7.4; 1M) was purchased from Applichem (Darmstadt, Germany). Standard chemicals and solvents, unless otherwise stated, were of analytical grade and were obtained from commercial sources; other reagents, chemicals and buffer salts were from Merck or Fluka AG.

**HPLC with radiodetection**: HPLC was performed using Agilent 1100 (Agilent Technologies, Waldbronn, Germany) liquid chromatography. The components were separated using a C18 column (Nucleosil 100 C18 Nautilus analytical column; 4.0 × 250 mm; Macherey-Nagel, Germany) protected by an 8.0 × 4.0 mm guard column of the same stationary phase and maintained at 40°C. Elution was performed with a gradient of 0.5% formic acid and 0.1% trifluoroacetic acid in water (mobile phase A) and 0.5% formic acid and 0.1% trifluoroacetic acid in acetonitrile (mobile phase B) at a flow rate of 1 mL/min. The following linear gradient was used: 0 min/40% B, 3 min/40% B, 5 min/45% B, 20 min/55% B, 22 min/100% B; 30 min/100% B, 30.5 min/40% B, 36 min/40% B. For online radioactivity detection, the effluent was mixed with the Rialuma^®^ liquid scintillation mixture (Lumac, Groningen, The Netherlands) at a flow rate of 3 mL/min. Radioactivity was detected on an HPLC radioactivity monitor equipped with a 0.5 mL flow cell (Berthold Technologies, Bad Wildbad, Germany).
