# Supplementary material for: In vitro studies and in silico predictions of fluconazole and CYP2C9 genetic polymorphism impact on siponimod metabolism and pharmacokinetics
Source: Eur J Clin Pharmacol. 2017 Dec 22;74(4):455–64. doi: 10.1007/s00228-017-2404-2 (PMC5849655; doi:10.1007/s00228-017-2404-2)
Supplement: Supplementary file 2 — (DOCX 20 kb) [file 228_2017_2404_MOESM2_ESM.docx]

**Supplementary materials**

**Online Resource 2.** SimCYP input parameters for siponimod

| **Input parameters** | **Siponimod (V9)** | **Siponimod (V12)** | **Siponimod (V16)** |
| --- | --- | --- | --- |
| Molecular weight (MW), (g/mol) | 516.61 | 516.61 | 516.61 |
| Log P: Octanol-water partition | 1.8 | 1.8 | 1.8 |
| Compound type | Ampholyte | Ampholyte | Ampholyte |
| pKa 1 | 3.3 | 3.3 | 3.1 |
| pKa 2 | 8.6 | 8.6 | 8.1 |
| Blood to plasma drug concentration ratio, B/P | 0.765 | 0.765 | 0.765 |
| Haematocrit (%) | 48 | 45 | 45 |
| Fraction unbound in plasma (fu) | 0.0002 | 0.0002 | 0.0002 |
| Main plasma binding protein | HSA | HSA | HAS |
| Q (gut) | 15.097 | 15.293 | 9.851 |
| fu (gut) | 0.0002 | 0.0002 | 0.0002 |
| Fraction available from dosage form, fa (%CV) | 1 | 1 | 0.91 (8.6) |
| Absorption rate constant, ka (%CV) (1/h) | 0.98 | 0.98 | 0.687 (7.8) |
| fg | 1 | 1 | 1.00 |
| Caco 2 permeability, P Caco-2 (10^-6^cm/s) | 16 | 16 | NA |
| PAMPA permeability (10^-6^ cm/s) | NA | NA | 10 |
| Reference compound permeability  (10^-6^cm/s) | Propranolol  5.33 | Propranolol  5.33 | NA |
| Active uptake into hepatocytes | 1 | 1 | 1 |
| Distribution | Minimal PBPK | Minimal PBPK | Full PBPK |
| Volume of distribution, Vss (L/kg) | 2.12 | 2.12 | 1.45 |
| Coefficient of variation, CV Vss (%) | 30 | 30 | 3.4 |
| *In vivo* iv clearance, CL_iv_ (L/h) | NA | NA | 3.12 |
| *In vivo* po clearance, CL_po_ (L/h) | 3.75 (mean) | 3.56 (median) | 3.71 |
| Total hepatic metabolic clearance in HLM, CL_int,_ (HLM) (µl/min/mg) | 3.8 | 3.8 | NA |
| fumic HLM | 0.017 | 0.017 | NA |
| *In vitro* intrinsic clearance, CL_int_ (CYP3A4) (µL/min/pmol, retrograde model fm) | 6.8  (18.5 %) | 6.108  (18.5 %) | 5.607  (18.5 %) |
| *In vitro* intrinsic clearance, CL_int_ (CYP2C8, µL/min/pmol)  (retrograde model fm) | 3.57  (1.7 %) | 3.204  (1.7 %) | 2.941  (1.7 %) |
| *In vitro* intrinsic clearance, CL_int_ (CYP2C19, µL/min/pmol)  (retrograde model fm) | 0.36  (0.1 %) | 0.323  (0.1 %) | 0.593  (0.1 %) |
| *In vitro* intrinsic clearance, CL_int_ (CYP2B6, µL/min/pmol)  (retrograde model fm) | 1.19  (0.4 %) | 1.064  (0.4 %) | 0.733  (0.4 %) |
| *In vitro* intrinsic clearance, CL_int_ (allelic CYP2C9*1*1, µL/min/pmol)  (retrograde model fm) | 54.67  (79.2 %) | 49.07  (79.2 %) | 45.105  (79.2 %) |
| *In vitro* intrinsic clearance, CL_int_ (allelic CYP2C9*1*2, µL/min/pmol,) retrograde model, extrapolated using *in vitro* result) | - | 33.03 | 45.885 |
| *In vitro* intrinsic clearance, CL_int_ (allelic CYP2C9*1*3, µL/min/pmol), retrograde model, extrapolated using *in vitro* result) | - | 26.72 | 24.605 |
| *In vitro* intrinsic clearance, CL_int_ (allelic CYP2C9*2*2, µL/min/pmol), retrograde model, extrapolated using *in vitro* result) | - | 16.93 | 33.271 |
| *In vitro* intrinsic clearance, CL_int_ (allelic CYP2C9*2*3, µL/min/pmol) retrograde model, extrapolated using *in vitro* result) | - | 10.65 | 18.92 |
| *In vitro* intrinsic clearance, CL_int_ (allelic CYP2C9*3*3, µL/min/pmol) retrograde model, extrapolated using *in vitro* result) | - | 4.37 | 2.869 |
| fumic *in vitro* (all enzymes) | 1 | 1 | 1 |
| Additional undefined HLM CL_int,_ (%CV) (µL/min/mg) | 0 | 0 | 0 |
| Non metabolic unbound CL_int_ (L/h) | 0 | 0 | 0 |
| Renal clearance in a 20−30 yr healthy male, CL_R_ (L/h) | 0 | 0 | 0 |
| Competitive inhibition constant, K_i_ CYP3A4/5 (µM) | 100 (IC50) | 100 (IC50) | - |
| Fraction unbound *in vitro*, fumic (CYP3A4) (microsomes) | 0.017 | 0.017 | - |
| Competitive inhibition constant, K_i_  (CYP3A5) (µM) | - | - | - |
| Fraction unbound *in vitro,* fumic (CYP3A5) | - | - | - |

*All siponimod values are from Novartis internal in vitro and in vivo studies.*

*CYP, cytochrome P450;* *fumic, fraction unbound in microsomes; HLM, human liver microsome; iv, intravenous; po, per oral; V, version*
