# Supplementary material for: In vitro studies and in silico predictions of fluconazole and CYP2C9 genetic polymorphism impact on siponimod metabolism and pharmacokinetics
Source: Eur J Clin Pharmacol. 2017 Dec 22;74(4):455–64. doi: 10.1007/s00228-017-2404-2 (PMC5849655; doi:10.1007/s00228-017-2404-2)
Supplement: Supplementary file 3 — (DOCX 20 kb) [file 228_2017_2404_MOESM3_ESM.docx]

**Supplementary materials**

**Online Resource 3.** SimCYP input parameters for fluconazole

| **Input parameters** | **Fluconazole (V9)*** | **Fluconazole (V16)*** |
| --- | --- | --- |
| Molecular weight, MW (g/mol) | 306.3 | 306.3 |
| Log P: Octanol-water partition | 0.2 | 0.2 |
| Compound type | monoprotic base | monoprotic base |
| pKa 1 | 1.76 | 1.76 |
| pKa 2 |  |  |
| Blood to plasma drug concentration ratio, B/P | 1 | 1 |
| Haematocrit (%) | 45 | 45 |
| Fraction unbound in plasma (fu) | 0.89 | 0.89 |
| Main plasma binding protein | HSA | HSA |
| Q (gut) | 12.6 | 14.376 |
| fu (gut) | 0.89 | 0.89 |
| Fraction available from dosage form, fa | 1 | 0.988 |
| Absorption rate constant, ka (1/h) | 1.28 | 1.863 |
| Caco 2 permeability, P Caco 2  (10^-6^cm/s) | 29.8 | 29.8 |
| Reference compound permeability  (10^-6^cm/s) | - | Multiple, scalar:  0.885 |
| Active uptake into hepatocytes | 1 | 1 |
| Volume of distribution, Vss (L/kg) | 0.57 | 0.748 |
| Coefficient of variation, CV Vss (%) | 30 | 30 |
| *In vivo* iv clearance, CL_iv_ (L/h) | 1.3 (SimCYP V7) | 1.01 |
| CV (%) CL_iv_ | 24 | 24 |
| *In vivo* po clearance, CL_po_ (L/h) | 1.5 | NA |
| Renal clearance in a 20-30 yr healthy male, CL_R_ (L/h) | 0.865 | 0.700 |
| Competitive inhibition constant, K_i_ (CYP2C19) (µM) | 2 | 2 |
| Fraction unbound *in vitro,* fumic (CYP2C19) | 1 | 1 |
| Competitive inhibition constant, K_i_ CYP2C9 (µM) | 7.92 | 20.4** [18] |
| Fraction unbound *in vitro*, fumic CYP2C9 | 1 | 1 |
| Competitive inhibition constant, K_i_ CYP3A4/5 (µM) | 10.7 | 10.7 |
| Fraction unbound *in vitro*, fumic (CYP3A4) (microsomes) | 1 | 1 |
| Competitive inhibition constant, K_i_ (CYP3A5) (µM) | 84.6 | 84.6 |
| Fraction unbound *in vitro,* fumic (CYP3A5) | 1 | 1 |
| Is the compound a MBI? | no | no |
| Is the compound a CYP inducer? | no | no |

**All values are from SimCYP version 9 and 16 (SimCYP Ltd, Sheffield, UK) unless specified otherwise; **Ki value adapted according to Neal et al [18] and a model verified by Certara (unpublished data).*

*CYP, cytochrome P450;* *fumic, fraction unbound in microsomes; MBI, mechanism based inactivation; V, version*
