# Supplementary material for: In vitro studies and in silico predictions of fluconazole and CYP2C9 genetic polymorphism impact on siponimod metabolism and pharmacokinetics
Source: Eur J Clin Pharmacol. 2017 Dec 22;74(4):455–64. doi: 10.1007/s00228-017-2404-2 (PMC5849655; doi:10.1007/s00228-017-2404-2)
Supplement: Supplementary file 4 — (DOCX 24 kb) [file 228_2017_2404_MOESM4_ESM.docx]

**Supplementary materials**

**Online Resource 4.** Additional methods on SimCYP PBPK models development.

In this paper we report the effect of CYP2C9 genetic polymorphism and inhibition effects from *in vitro* to *in silico* predictions with a history over several years. The fluconazole DDI simulation was done in 2010 using SimCYP PBPK version 9 to help with the planning of a clinical trial protocol. The predicted result was confirmed with the clinical DDI data in 2011. Two years later, the genetic polymorphism information was needed for the drug development, so the PG model with SimCYP version12 was developed, resulting in the prediction released in 2013. Our prediction on PG was confirmed in 2015 by the actual *in vivo* clinical results. The last simulation was done early this year with Version 16 to support the new drug application.

Because the Certara/SimCYP company releases a new version of the software every year and our studies were performed over several years, three versions of software were used,

Two different approaches were compared in this study to build the siponimod SimCYP model. In the first approach, the model was established based on physicochemical data, absorption data (ka, fa, fu[gut]) estimated based on preclinical information, distribution data coming from a minimal PBPK model of SimCYP, and HLM metabolic CL_int_ data of different CYP2C9 genotypes to account for the clearance differences. The fractional contribution of the involved CYP enzymes was estimated based on the data of the recombinant CYP enzyme incubations. Using the mean and median clinical clearance values of siponimod after oral single dosing in the dose ranged from 0.1 mg to 75 mg, the respective CL_int_ values for each CYP enzyme were calculated by a retrograde model approach. In a separate *in vitro* experiment the metabolism activities for siponimod were determined using single donor HLM of the genotypes CYP2C9*1/*1, *2/*2 and *3*/3*. These CL_int_ values were extrapolated by linear scaling to the heterozygote genotypes and pharmacogenetics ratios (PG ratios) were calculated (Table 1). Applying these PG ratios to the CYP2C9 CL_int_, genotype specific Cl_int_ values were calculated, which were applied to the SimCYP model. This model was verified with clinical data from several single dose studies that predicted the DDI effect of fluconazole and a genotype PK study (CYP2C9*1/*1, *2/*3 and *3/*3) (Ref 19, companion manuscript EJCP-D-17-00482, Gardin et al). The second approach was based on physicochemical data, absorption data (ka [PopPK], fa [hADME], fu[gut] estimated based on C_max_ DDI sensitivity towards fluconazole), distribution data from a clinical i.v. study. Using the clinical i.v. clearance data of siponimod the respective CL_int_ values for each CYP enzyme were calculated by a retrograde approach. The fractional contribution of the involved CYP enzymes was estimated based on the data of the recombinant CYP enzyme incubations. The PopPK model delivered genotype-specific CL/F values, which were converted to CYP2C9-specific hepatic clearance values for each genotype using absolute bioavailability and the fractional CYP2C9 contribution. In the final step these clearances were converted to intrinsic CYP2C9 CL_int_ values for each genotype (**Table 6**). For building this model, the clinical data of the following studies were used: single and multiple ascending dose, fluconazole DDI, absolute bioavailability study. The model was verified with clinical data (Ref 19, companion manuscript EJCP-D-17-00482, Gardin et al) of a genotype PK study (CYP2C9*1/*1, *2/*3 and *3/*3) and the rifampicin DDI study.

###### **Table 6.** Intrinsic clearance values of allelic CYP2C9 genotypes calculated based on genotype specific clearance data from PopPK analysis

| **Genotype** | **CL (L/h)** | **CL driven by CYP2C9 (L/h) ^1^** | **CL_int_ CYP2C9*1/*1 (μL/min/pmol)^3^** | **CL_int_,CYP2C9 multiplied by Factor A ^2^** | **relative contribution compared to CL_int_,2C9*1/*1** | **CL_int_,CYP2C9 (μL/min/pmol)** |
| --- | --- | --- | --- | --- | --- | --- |
| *1/*1 | 3.12 | 2.47416 | 45.1049 | 0.62123 | 1 | 45.1049 |
| *1/*2 | 3.16056 | 2.51472 |  | 0.631979 | 1.017303 | 45.88537 |
| *1/*3 | 2.028 | 1.38216 |  | 0.338881 | 0.545501 | 24.60475 |
| *2/*2 | 2.496 | 1.85016 |  | 0.458245 | 0.737642 | 33.27126 |
| *2/*3 | 1.716 | 1.07016 |  | 0.260633 | 0.419544 | 18.92348 |
| *3/*3 | 0.8112 | 0.16536 |  | 0.039508 | 0.063597 | 2.868516 |

^1^Calculation of CL driven by CYP2C9: CLp(CYP2C9) = total CLp –total CLp*0.207, with fm,CYP2C9: 0.793 and fm,other: 0.207, with CLp as plasma clearance

^2^CL_int_,CYP2C9 calculated by solving well-stirred model equation: CL_int_,CYP2C9 * Factor A = CLp(CYP2C9)*Qp / (Qp-CLp(CYP2C9)), with CLp and Qp as plasma clearance and liver plasma flow, respectively. Factor A correction factor accounting for the product of fup/fumic*hepatic enzyme abundance*scaling factor to account for unit change

^3^CL_int_,CYP2C9 calculated by retrograde calculator in SimCYP

CL, clearance; CL_int_, intrinsic clearance
